# Zinc accumulation in the jaw of *Nereis aibuhitensis*

**DOI:** 10.1242/jeb.251316

**Published:** 2026-04-16

**Authors:** Yugo Kato, Wataru Kashiwabara, Mayumi Iijima, Keisuke Shimizu, Lumi Negishi, Hitoshi Kurumizaka, Yu Maekawa, Takenori Sasaki, Akiko Hokura, Shino Homma-Takeda, Michio Suzuki

**Affiliations:** ^1^ Department of Applied Biological Chemistry, Graduate School of Agricultural and Life Sciences, The University of Tokyo, 1-1-1 Yayoi, Bunkyo-ku, Tokyo 113-0032, Japan; ^2^ Institute for Radiological Science, National Institutes for Quantum Science and Technology, 4-9-1 Anagawa, Inage-ku Chiba 263-8555, Japan; ^3^ Institute for Quantitative Biosciences, The University of Tokyo, 1-1-1 Yayoi, Bunkyo-ku, Tokyo 113-0032, Japan; ^4^ The University Museum, The University of Tokyo, Tokyo 113-0033, Japan; ^5^ Department of Applied Chemistry, School of Engineering, Tokyo Denki University, Tokyo 120-8551, Japan

**Keywords:** Biomineralization, Biomechanics, Green worm

## Abstract

Zinc accumulates in the jaws of green worms, *Nereis aibuhitensis*, a phylum of annelid worms, to enhance the mechanical properties of the jaws for predation and migration. In this study, we precisely mapped the localization of zinc and identified the matrix proteins responsible for its binding in the jaws. X-ray diffraction analysis revealed no distinct crystalline peaks in powdered jaw samples, indicating that zinc exists predominantly in a non-crystalline form. X-ray absorption fine structure spectra further demonstrated that zinc coordinates with organic molecules containing imidazole groups, implicating histidine (His) residues in zinc binding. Elemental analysis by inductively coupled plasma mass spectrometry and particle-induced X-ray emission showed zinc was concentrated on the inner side of the jaw tip, while halogens were mainly localized on the outer surfaces of the jaw. A comparison of protein extracts from the zinc-rich jaw tip and the zinc-poor bottom showed a specific protein band at the tip region, identified as a His-rich protein (Nai11527). These findings reveal a previously uncharacterized mechanism whereby histidine-rich proteins bind zinc to reinforce jaw structure. This study advances our understanding of biomineralization and offers a promising blueprint for the design of novel bio-inspired materials with enhanced mechanical properties.

## Teaser

**Summary:** Histidine-rich proteins in *Nereis aibuhitensis* jaws bind zinc in non-crystalline form, enhancing jaw mechanical strength by a previously uncharacterized mechanism.

## INTRODUCTION

Many organisms possess hard tissues, such as teeth, in their mouths to aid in feeding. These structures must withstand high forces during predation and, therefore, require significant mechanical strength. In humans and other higher organisms, teeth are primarily composed of calcareous material. In contrast, the jaws and beaks in several species are mainly composed of organic molecules (Miserez et al., 2023). Several animal groups – including Chordata, Arthropoda, Onychophora, Annelida and Gnathostomulida – possess specialized jaws. These jaws are structured to open and close for effective prey capture and are also used for grasping or manipulating objects for movement or other activities. Arthropods and annelids often incorporate metal elements such as iron (Fe), copper (Cu), zinc (Zn) and manganese (Mn) into their jaws, enhancing hardness to levels comparable to those of calcified tissue (Schofield, 2005). For instance, the jaws of the ragworm (phylum Annelida, class Polychaeta) are enriched with various metals, which vary by species. While around 12,000 species of ragworm are known, it is estimated that a further 6000 are yet to be discovered (Appeltans et al., 2012). Because of their diverse feeding habits and ecological niches, different metals are believed to be concentrated in their jaw regions. Such metal enrichment is widely observed across invertebrates; for example, Cu-based atacamite [Cu_2_(OH)_3_Cl] in the jaws of *Glycera dibranchiata*, Zn in *Nereis limbata* and several insects such as crickets and *Atta cephalotes*, and Fe or Ca in the radulae of mollusks (Birkedal et al., 2006; Lichtenegger et al., 2002; Schofield et al., 2021; Weaver et al., 2010; Xing et al., 2013).

**Table:** 

**List of abbreviations**	
Br	bromine
Cl	chlorine
CT	computed tomography
*E*_IT_	Young's modulus value
EDS	energy-dispersive spectroscopy
Fe	iron
*H*_IT_	hardness value
His	histidine
I	iodine
ICP-MS	inductively coupled plasma mass spectrometry
Mn	manganese
PIXE	particle-induced X-ray emission
SDS-PAGE	SDS-polyacrylamide gel electrophoresis
SEM	scanning electron microscope
XAFS	X-ray absorption fine structure spectra
XRD	X-ray diffraction
Zn	zinc

These transition metals such as Zn, Cu, Mn and Fe greatly increase the hardness and stiffness of biological tissues. For instance, Zn-rich jaws of leaf-cutter ants and venom-injecting forcipules of centipedes, Cu-containing jaws of bloodworms and Mn-enriched stings of scorpions exhibit mechanical properties approaching or exceeding those of enamel and nacre (Birkedal et al., 2006; Lichtenegger et al., 2002; Schofield, 2005; Schofield et al., 2021; Züger et al., 2024). A strong correlation between Zn concentration and hardness has been experimentally demonstrated in the mandibles of the leafcutter ant (Birkenfeld et al., 2024). The types of transition metals enriched in mouthparts vary depending on feeding habits (Sevarika and Romani, 2024).

Green worms, a species of ragworm, live under the soil in the ocean and brackish estuarine areas. Green worms feed on various organisms, including predators of small animals, seagrasses and organic matter, by swallowing sand and mud. Green worms also have hard jaws that contain Zn (Broomell et al., 2006; Schofield et al., 2021). We explored the chemical form of Zn and the mechanism of Zn concentration in the hard jaws. We focused on the green worm *Nereis aibuhitensis*, which is easy to obtain as fishing bait in Japan and convenient to handle because of its small body size. Zn and chlorine (Cl) seem to coordinate directly with proteins. The chemical form of Zn in jaws has been hypothesized as a model of Zn ion coordination to a histidine-rich protein. For instance, zinc coordination with histidine residues contributes to cuticular stiffening in green worms *Nereis virens* (Khare et al., 2023). However, the molecular basis of zinc incorporation in *N. aibuhitensis* remains unexplored. In this study, we analyzed the chemical form and localization of Zn in the jaw of green worms and the proteins associated with Zn.

## MATERIALS AND METHODS

### Strain identification

We bought green worms farmed in Osaka, Japan. DNA was extracted from the green worm to identify the species. Living tissue was cut out using scissors and centrifuged. The resulting pellet of living tissue was washed and centrifuged twice with phosphate-buffered saline. The precipitation was mixed with 50 µl of TE [10 mmol l^−1^ Tris-HCl (pH 8.0), 1 mmol l^−1^ EDTA (pH 8.0)], 500 µl of extraction buffer [200 mmol l^−1^ NaCl, 10 mmol l^−1^ Tris-HCl (pH 8.0), 10 mmol l^−1^ EDTA (pH 8.0), 1% SDS] and 5 µl of 5 mg ml^−1^ proteinase K solution. The mixture was incubated at 55°C for 3 h. Following addition of an equal volume of PCI solution (phenol:chloroform:isoamyl alcohol 25:24:1 v/v), the suspension was mixed using a vortex mixer and rotating mixer for 10 min at room temperature. The mixture was centrifuged at 22,000 ***g*** for 10 min and the supernatant was collected in a plastic tube. PCI solution was added to the precipitate and the suspension was mixed and centrifuged similarly. The supernatant was collected in the same plastic tube, mixed with an equal volume of chloroform and centrifuged at 22,000 ***g*** for 10 min. The upper layer was mixed with isopropanol (0.7 times the volume of the upper layer) and centrifuged at 22,000 ***g*** for 10 min. Following removal of the supernatant, the precipitate was mixed with 1 ml of 70% ethanol and centrifuged at 22,000 ***g*** for 10 min. The precipitate was semi-dried and dissolved in 10 µl of TE. To degrade RNA, 1 µl of 10 mg ml^−1^ RNase A was added, and the mixture was incubated at 37°C for 30 min. The extracted DNA was used to amplify the *16S* rRNA gene by PCR. PCR was performed in a final volume of 10 μl containing 0.05 μl TaKaRa Ex Taq, 1 μl 10× Ex Taq Buffer, 0.8 μl dNTP mixture, 1.0 μl each primer, 1 μl DNA and 5.15 μl deionized water. The primers used were *16S* rRNA gene primers 16SbrH (5′CCGGTCTGAACTCAGATCACGT 3′) and 16SarL (5′CGCCTGTTTATCAAAAACAT 3′) (Tosuji and Sato, 2012). The reaction mixture was subjected to 35 amplification cycles as follows: denaturation at 96°C for 30 s, annealing at 58°C for 30 s and extension at 72°C for 1 min 30 s. For purification, the PCR products were subjected to ethanol precipitation. The purified PCR products were sequenced using each primer (16SbrH and 16SarL) and the ABI3730xl DNA Analyzer (Applied Biosystems). The *16S* rRNA sequences of the *Nereis*, *Neanthes* and *Glycera* species were obtained from NCBI using a BLAST search (Table S1). The multiple alignment of these sequences was conducted using MAFFT (v.7, https://mafft.cbrc.jp/alignment/server/index, last accessed 16 May 2025; Katoh et al., 2019). Phylogenetic analysis was performed by the maximum likelihood method using MEGA (Molecular Evolutionary Genetics Analysis) (v.11) (Tamura et al., 2011) and the online version of RAxML (Stamatakis, 2014), respectively.

### Scanning electron microscope observation and energy-dispersive spectroscopy

The untreated jaw and the cross-section of the jaw were observed using a scanning electron microscope (SEM). To make a cross-section, the jaw was embedded in epoxy resin by curing in a mixture of EpoxiCure 2 Epoxy Resin and EpoxiCure 2 Epoxy Hardener (Buehler, Lake Bluff, IL, USA) at a volume ratio of 4:1 for 24 h. Resin sections were prepared by cutting with IsoMet LS (Buehler) and polished using and EcoMet30 polisher (Buehler). The polished sample was rinsed with deionized water and stored in a desiccator until measurement. SEM observation was conducted using an S-4800 SEM (Hitachi, Tokyo, Japan) with a cold field-emission gun at 15 kV. Samples were coated with platinum and palladium before observations using an ion sputtering device (E-1030, Hitachi). The elemental composition was analyzed by X-ray microanalysis by energy-dispersive spectroscopy (EDS) using an X-ray analyzer (EMAX, Horiba, Kyoto, Japan) mounted on the SEM.

### Micro-computed tomography observation

3D visualization of the jaw was carried out using a microfocus X-ray computed tomography (CT) scanner (ScanXmate-B100TSS110, Comscan techno, Kanagawa, Japan) with high-resolution settings (voltage 70 kV, detector array size 1024×1012 pixels, 360 deg rotation, 1200 projection). Geometric resolution varied from 2 to 4 μm depending on the size of each shell. The acquired slice data were rendered as 3D images using a 3D analysis suite (ConeCTexpress; WhiteRabbit Corp., Tokyo, Japan). 2D images were obtained from the 3D data, and ImageJ v.1.50i (Schneider et al., 2012) was used to measure sample size.

### Measurement of elemental concentration in the jaw by inductively coupled plasma mass spectrometry

Each of the 15 jaws was divided into three equal parts: tip, middle and root. Each sample and 4.00 mg of soft tissue as a control were decomposed with nitric acid. After adding 1 ml of concentrated nitric acid, wet ashing decomposition was carried out at 150°C for 12 h in PTFE containers. The decomposed material was dissolved and made up to 5 ml volume with 0.8 mol l^−1^ nitric acid solution. The dilution was filtered (0.45 µm filter; 25AS045AS, Advantec, Tokyo, Japan) and subjected to inductively coupled plasma mass spectrometry (ICP-MS; 7800 ICP-MS, Agilent Technologies, CA, USA). ICP-MS was used to measure the metal concentrations under conditions Cl (with *m*/*z*=35), Zn (with *m*/*z*=66), Br (with *m*/*z*=81) and I (with a *m*/*z*=127).

### Micro-particle-induced X-ray emission analysis

For measurement of elemental distribution, micro-particle-induced X-ray emission (PIXE) measurements were taken of the entire jaw and cross-section of the jaw. Micro-PIXE measurements were carried out at the PIXE Analysis System in the Tandem Accelerator at the National Institute for Quantum Science and Technology, Chiba, Japan, using a micro-PIXE system (Model OM2000, Oxford Microbeams Ltd, Oxford, UK) with a Si(Li) detector (Homma-Takeda et al., 2012). Two-dimensional analysis was conducted as follows: proton energy, 2.6 MeV; integrated current, 0.20 μC; beam size, 1 μm×1 μm; scan size, 500×500 μm^2^. The OMDAQ data acquisition system (Oxford Microbeams Ltd) was used for analysis. The sample scanning areas were 256×256 steps in 1 mm^2^. For elemental mapping, Zn *K*α (8.491–8.762 keV), Cl *K*α (2.545–2.711 keV), Br (bromine) *K*α (11.773–12.074 keV) and I (iodine) *L*α (3.840–4.035 keV) were measured.

### Measurement of physical properties

The physical properties of the jaws were assessed using a microcompression testing machine (MCT-510, Shimadzu, Kyoto, Japan) with a Berkovich diamond probe tip (triangular pyramid with a tip angle of 115 deg and a tip radius of up to 0.1 µm). The jaw sample was embedded in resin, sectioned, and stored in a desiccator until measurement. The test was performed in a load–hold–unload mode at room temperature. The loading and unloading rates were each 0.49 mN s^−1^, with a 5 s hold segment at the maximum load (100 mN) and an additional 5 s hold after unloading. The indentation hardness (*H*_IT_) and Young's modulus values (*E*_IT_) were calculated automatically.

### X-ray diffraction measurement

The jaw of the green worm was ground using a mortar and pestle. The powdered jaw was placed in a reflection-free sample holder. X-ray diffraction (XRD) patterns were collected using a RINT-Ultima^+^ diffractometer (Rigaku, Tokyo, Japan) with Cu *K*α radiation emitted at 40 kV and 30 mA. A 10 mm divergence slit, a 5 mm mask to confine the beam width and an 8 mm anti-scatter slit were applied. The data were collected at 0.5 deg intervals with a scan rate of 0.01 deg min^−1^.

### X-ray absorption fine structure measurement

Zn K-edge X-ray absorption fine structure (XAFS) measurements were carried out at BL-9A (Photon Factory, Institute of Materials Structure Science, High Energy Accelerator Research Organization, Tsukuba, Japan). X-ray absorption spectra were measured in transmission mode for the pellet samples or fluorescence mode for liquid samples in a mylar membrane by detecting X-ray fluorescence intensities using a 19-element Ge solid-state detector (SSD). As pellet samples, green worm jaw, ZnO, Zn(OH)_2_, ZnCl_2_ and ZnBr_2_ were prepared. The samples were mixed with an appropriate amount of boron nitride and ground using a mortar and pestle (Table S2). The pellet samples were prepared from mixed powder using a tablet molding machine (Shimadzu). As a liquid sample, 13.6 mg of imidazole and 3.28 mg of ZnSO_4_ were dissolved in 100 ml of distilled water; 100 µl of imidazole-Zn solution was sealed with a Mylar film (Chemplex Industries, Palm City, FL, USA).

### SDS-PAGE

To identify the protein binding to Zn, jaws were analyzed by SDS-polyacrylamide gel electrophoresis (SDS-PAGE). Twenty jaws of green worms (approximately 15 mg) were divided into tips and roots. Each part was ground using a pestle and a mortar. The samples were soaked in 300 µl of 5% CH_3_COOH and 8 mol l^−1^ urea solution for 24 h to extract protein. Each extraction was mixed with an equal volume of sample buffer [0.125 mol l^−1^ Tris-HCl buffer (pH6.8), 10% 2-mercaptoethanol, 4% SDS, 10% sucrose, 0.01% Bromophenol blue], heated at 100°C for 5 min and applied to the SDS-PAGE gel (12%). Following electrophoresis, the gel was silver stained using SilverQuest™ Staining Kit (Thermo Fisher Scientific, Waltham, MA, USA).

### RNA sequencing

The soft tissue of the green worm cut by scissors was stirred with 1 ml of Sepasol^®^ RNA I Super G by a vortex mixer and allowed to react for 5 min. The sample was treated with 200 µl of chloroform for 3 min after inversion and centrifuged (12,000 ***g***, 4°C, 15 min). The upper layer was treated with 500 µl of 2-propanol for 10 min and the supernatant was removed. The precipitate was suspended in 1 ml of 75% ethanol and the supernatant removed. After air drying, the precipitate was dissolved in TE buffer and sent to BGI Japan (Hyogo, Japan) for transcriptome sequencing. Polyadenylated RNA was enriched using oligo(dT) beads and subsequently fragmented. First- and second-strand cDNA synthesis was performed using N6 random primers. After end-repair, A-tailing and adaptor ligation, the cDNA libraries were amplified by PCR. The resulting single-stranded DNA molecules were circularized and sequenced on the DNBSEQ platform (MGI, Shenzhen, China) with paired-end 150 bp reads. Clean reads were obtained by removing low-quality reads, adapter contamination and reads containing more than 5% unknown bases (*N*). *De novo* transcriptome assembly was performed using Trinity v.2.0.6, followed by clustering using Tgicl v.2.0.6 to generate a non-redundant set of unigenes.

### LC-MS/MS analysis

The specific band at the tip in SDS-PAGE was separated by a razor and destained with SilverQuest™ (Thermo Fisher Scientific) following the manufacturer's protocol. The destained gel was washed with 100 µl of acetonitrile for 15 min with tapping every 5 min and the supernatant was removed. After drying using a centrifugal evaporator, the gel was treated with 50 µl of 10 mmol l^−1^ DTT and 100 mmol l^−1^ NH_4_HCO_3_ for 1 h at 56°C and the supernatant was removed. The gel was alkylated with 50 µl of 55 mmol l^−1^ iodoacetamide and 100 mmol l^−1^ NH_4_HCO_3_ for 45 min at room temperature with tapping every 15 min in the dark and the supernatant was removed. The gel was treated with 100 µl of 100 mmol l^−1^ NH_4_HCO_3_ for 10 min, after which the supernatant was removed. The gel was then dehydrated with 100 µl of acetonitrile for 15 min, and the supernatant was removed. Finally, the gel was shaken with 100 µl of 100 mmol l^−1^ NH_4_HCO_3_ for 15 min using a micromixer, and the supernatant was removed. The gel was treated with 100 µl of acetonitrile for 15 min with tapping every 5 min and the supernatant was removed. After dying using a centrifugal evaporator for 10 min, the dried gel was treated with 10 µl of 10 ng µl^−1^ Trypsin Gold (Promega) in 50 mmol l^−1^ NH_4_HCO_3_ for 10 min and excess solution was removed. The gel was covered with ∼20 µl of 50 mmol l^−1^ NH_4_HCO_3_ for 15 h at 37°C. The supernatant was removed to a new tube. The gel was shaken with 40 µl of Milli-Q for 20 min using a micromixer and the supernatant was removed to the same tube. The gel was shaken with 20 µl of 60% acetonitrile containing 0.1% trifluoroacetic acid solution for 15 min by a micromixer, and the supernatant was removed to the same tube. This process was repeated using 80% acetonitrile containing 0.1% TFA and 100% acetonitrile containing 0.1% TFA. The tube containing the supernatant was centrifuged at 15,000 ***g*** for 5 min and the supernatant was removed, leaving ∼5 µl sample. The remaining sample was dried by a centrifugal evaporator. The dried sample was diluted with 20 µl of 0.1% TFA solution by micromixer and used for LC-MS/MS analysis (Orbitrap Velos, Thermo Fisher Scientific). The data from LC-MS/MS analysis were analyzed using the software Proteome Discover 2.1 (Thermo Fisher Scientific) and the predicted protein sequence from the transcriptome data.

## RESULTS

### Green worm species identification

**Fig. 1.**
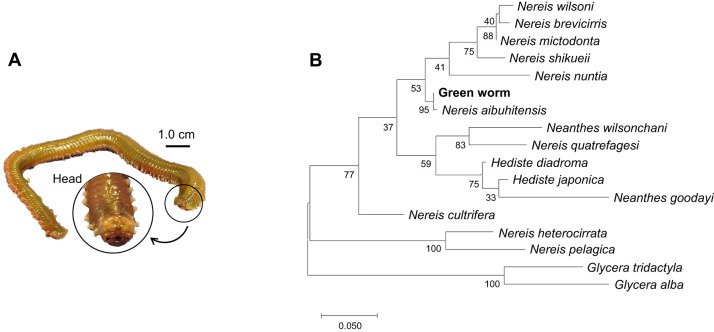
**Morphology and classification of green worms.** (A) Photo of a green worm. The head is circled and shown at higher magnification in the inset. (B) Phylogenetic tree analysis of green worms generated a phylogenetic tree by the maximum-likelihood method. Numbers at nodes indicate bootstrap values (%).

To identify the green worm species (Fig. 1A), molecular phylogenetic analysis was performed using the *16S* rDNA sequence. Phylogenetic trees by the maximum likelihood method are shown in Fig. 1B. *Glycera tridactyla* and *Glycera alba* of the family Tilariaceae were used as the outer group in this study. The phylogenetic tree demonstrated that the green worm was *Nereis aibuhitensis* (Grube 1878).

### Morphological observation of the jaw

**Fig. 2.**
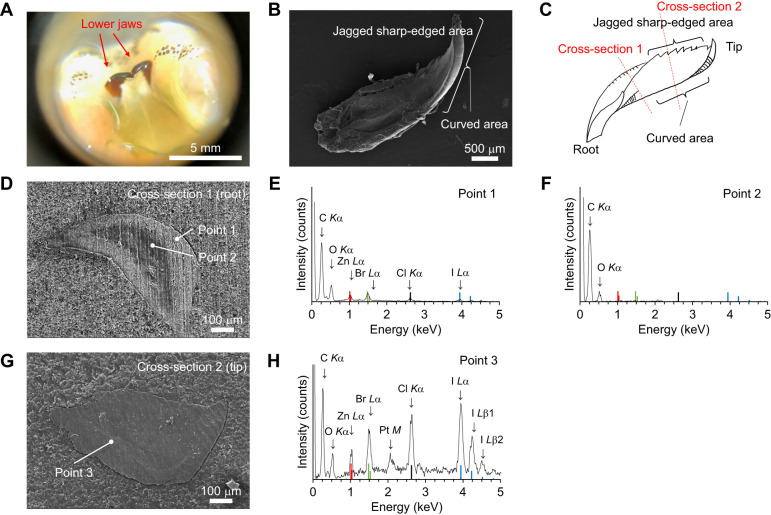
**Microscopic image and energy-dispersive spectroscopy (EDS) spectrum of the jaw.** (A) Microscope image of the green worm head. A pair of jaws is indicated by the red arrows. (B) Scanning electron microscope (SEM) image of the entire jaw. (C) Schematic model of the jaw. The tip is on the right and the root is on the left. The jaw was cut at the positions indicated by the red lines, and the cross-sections on the root (1) and tip (2) side were observed using the SEM. (D,G) SEM images of the cross-section at the position indicated by the red line in C (D: line 1, G: line 2). (E,F,H) EDS spectra of the lower jaw (E: point 1 in D; F: point 2 in D; H: point 3 in G). The vertical bars indicate the standard energies of characteristic X-ray emission lines for each element (red: Zn, black: Cl, green: Br, blue: I) according to Thompson and Vaughan (2001). Pt peak derived from the conductive coating.

Fig. 2A shows a picture of a green worm head, highlighting the pair of jaws. The whole jaw was observed by SEM imaging (Fig. 2B). The jaw was about 3 mm long and 1 mm wide, twisted towards the direction of growth. The jaw was jagged, sharp-edged medially and curved laterally. The resin cross-sections of the jaw were observed using SEM imaging. The cross-section from the root side showed that a dark central region was surrounded by bright areas. The thickness of the bright area was approximately 100 µm (Fig. 2D). The EDS spectrum indicated that the bright area contained metallic elements and halogens such as Zn, Br and Cl (Fig. 2E). In contrast, the central dark area consisted of C and O. These results revealed that organic molecules without metallic elements were in the center (Fig. 2F). Conversely, the contrast of the cross-section from the tip side was homogeneous (Fig. 2G). The EDS spectrum demonstrated that the cross-section was hard tissue containing metallic elements and halogens such as Zn, Br, Cl and I (Fig. 2H).

### 3D structure of the jaw

**Fig. 3.**
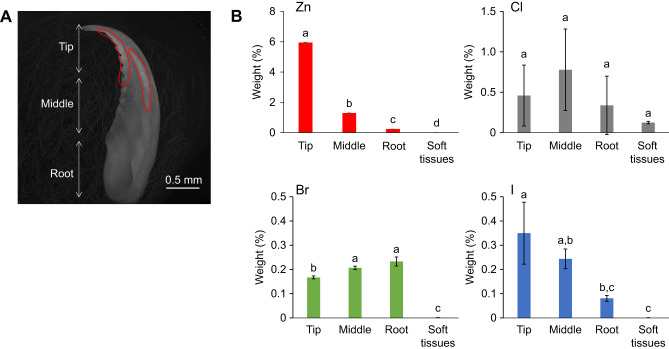
**Micro-computed tomography (CT) and elemental analysis of the jaw.** (A) Micro-CT images of the green worm jaw. The areas circled in red appear brighter and are thought to be clusters of elements with higher atomic numbers. (B) Weight percentage of Zn, Cl, Br and I in each part of the lower jaw and soft tissue. Statistical significance was determined using the Tukey–Kramer test and different letters indicate significant differences (*P*<0.05).

SEM and EDS analysis showed that the hard tissue of the jaw contained Zn and halogens. To visualize the localization of elements with large atomic numbers, the jaw was observed by micro-CT. The results are illustrated in Fig. 3A and Movie 1. In micro-CT observation, elements with large atomic numbers appeared in areas that appeared as bright contrasts. The jagged, sharp-edged area and curved area were bright. The tip did not show a bright contrast, indicating that there were no heavy elements in the tip.

### Elemental and physical properties of the jaw

The jaw was separated into three sections (tip, middle and root). The mass of elements (Zn, Cl, Br and I) in each section was measured by ICP-MS. The results are illustrated in Fig. 3B and Table 1. Zn was in low concentrations in the soft tissue. Among the three sections, the concentration of Zn increased from root to tip. Cl was present in the soft tissue, and the concentration of Cl did not differ significantly between any sections of the jaw. Br and I were rarely found in the soft tissue. Among the three sections, the concentration of Br at the root was the highest, while that of I was the highest at the tip.

**Table 1.** Weight (%) of Zn, Cl, Br and I in each of the lower jaw and soft tissue

	Zn	Cl	Br	I
Tip	5.95±0.02^a^	0.46±0.38 ns	0.17±0.01^b^	0.35±0.13^a^
Middle	1.30±0.01^b^	0.78±0.50 ns	0.21±0.01^a^	0.24±0.04^a,b^
Root	0.24±0.00^c^	0.34±0.36 ns	0.23±0.02^a^	0.08±0.01^b,c^
Soft tissues	0.01±0.00^d^	0.12±0.02 ns	0.00±0.00^c^	0.00±0.00^c^

Statistical significance was determined using the Tukey–Kramer test. Different letters indicate significant differences (*P*<0.05).

**Fig. 4.**
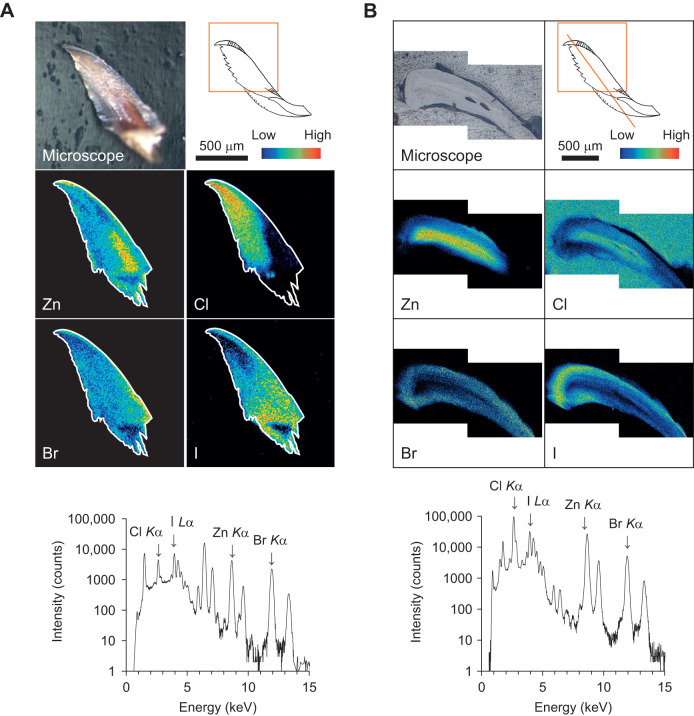
**Elemental mapping and characteristic X-ray spectrum by micro-particle-induced X-ray emission (PIXE) analysis.** (A) Entire jaw and (B) cross-section of the jaw. White lines in elemental mapping indicate the contour of the jaw. Intensity mapping is displayed only for the region inside the white lines. The spectra are characteristic X-ray spectra of each region. Micro-PIXE was performed in a 1500 µm×1500 µm area (A) or 1000 µm×1000 µm (B) area with 256×256 step scanning. Proton energy, 2.6 MeV; beam current, 10 pA; integrated current, 0.3 lC; spatial resolution, 1 µm×1 µm area.

To analyze detailed elemental distribution, micro-PIXE analysis of the whole jaw and a cross-section of the jaw was performed. From the elemental mapping of the entire intact jaw (Fig. 4A), Zn was more abundant at the tip and gradually decreased toward the root. Additionally, Zn was more prevalent in the center than at the edges. Conversely, halogens were localized in regions with a low concentration of Zn. The distribution of halogens also varied for each element. Cl was abundant on the inside near the root. Br was abundant on the outer edges. I was abundant on the outside near the root. The elemental mapping of the cross-section embedded in the resin also showed that Br and I were present in a region with low Zn concentration (Fig. 4B). In contrast, Cl localization correlated with high Zn concentration. The high Cl concentration regions around the sample denote high levels of Cl due to the embedded resin. Zn was abundant in the central part of the jaw and most abundant at the 100 µm distance from the jagged, sharp-edged area. Cl was also plentiful in the same region as Zn. In contrast, Br and I were concentrated in the outer jaw, the region with low Zn concentration.

**Fig. 5.**
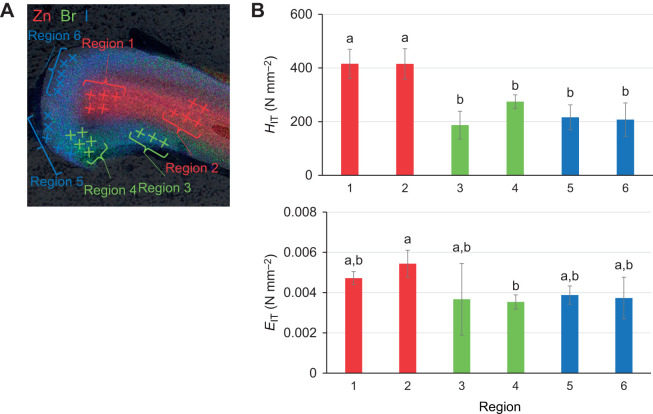
**Physical properties of the jaw.** (A) The area measured by nanoindentation. Different colors correspond to the different elements (red: Zn, green: Br, blue: I), and color intensity increases with increasing elemental concentration. (B) The nanoindentation hardness values (*H*_IT_) and Young's modulus values (*E*_IT_) of each region in A (*N*=5 indents; 1 specimen, means±s.d.). Region 1 and region 2 are Zn-concentrated regions. Region 3 and region 4 are Br-concentrated regions. Region 5 and region 6 are I-concentrated regions. Statistical significance was determined using the Tukey–Kramer test. Different letters indicate significant differences (*P*<0.05).

The hardness of the jaws was obtained by nanoindentation. The Zn- and Cl-concentrated regions, Br-concentrated regions and I-concentrated regions were each measured with reference to the elemental mapping obtained by micro-PIXE (Fig. 5A). The average of the nanoindentation hardness values (*H*_IT_) and Young's modulus values (*E*_IT_) at three points in each region are shown in Fig. 5B. There was no significant difference between the two Zn- and Cl-concentrated regions (regions 1 and 2) in either *H*_IT_ (415.5±54.1 and 415.0±56.8 N mm^−2^) or *E*_IT_ (0.00472±0.00033 and 0.00544±0.00067 N mm^−2^) values. The *H*_IT_ values of the Zn- and Cl-concentrated regions were greater than those of the Br-concentrated regions (regions 3 and 4; 186.8±51.6 and 274.4±25.4 N mm^−2^) and the I-concentrated regions (regions 5 and 6; 216.0±46.6 and 207.5±61.1 N mm^−2^). In regions 3 and 5, some load-displacement curves showed less ideal behavior. However, in regions 4 and 6, which showed similar Br or I concentration, *H*_IT_ values were comparable to those of regions 3 and 5. Overall, higher *H*_IT_ values were observed in the Zn- and Cl-concentrated regions.

To further examine the variation in mechanical properties along the jaw, *H*_IT_ measurements were performed for two additional regions near the root, in addition to the two tip regions described above. Statistical analysis revealed that *H*_IT_ values were significantly higher in the tip-side regions than in the root-side regions (Fig. S1). Representative load–displacement curves corresponding to Fig. 5 and Fig. S1 are shown in Fig. S2.

### The chemical form of Zn in the jaw

**Fig. 6.**
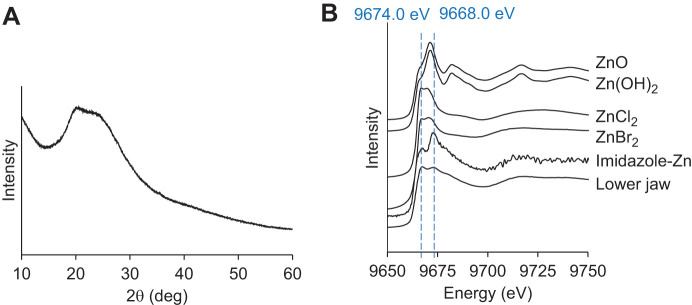
**X-ray diffraction (XRD) and X-ray absorption fine structure spectra (XAFS) analysis of the jaw.** (A) XRD pattern of the powdered lower jaw of the green worm. The horizontal axis represents 2θ, the diffraction angle. (B) XAFS spectra of the jaw of the green worm and controls [ZnO, Zn(OH)_2_, ZnCl_2_, ZnBr_2_ and Zn bound to an amino group in imidazole]. Peaks appeared at 9668.0 and 9674.0 eV for the jaw, indicated by the blue dashed lines.

To reveal the chemical form of Zn in the jaw, the jaw was subjected to XRD and XAFS analysis. As no sharp peak was observed in the XRD pattern of the powdered jaw, Zn was not present in the form of mineral crystals (Fig. 6A). XAFS spectra are shown in Fig. 6B. Standard spectra were measured for ZnO, Zn(OH)_2_, ZnCl_2_, ZnBr_2_ and Zn bound to an amino group in imidazole. There were two peaks at 9668.0 eV and 9674.0 eV in the jaw. Although there was no exact match to the jaw peaks in the controls, the jaw was close to Zn bound to the amino group in imidazole and zinc halide in peak position and peak size.

### Identification of the Zn-binding protein

**Fig. 7.**
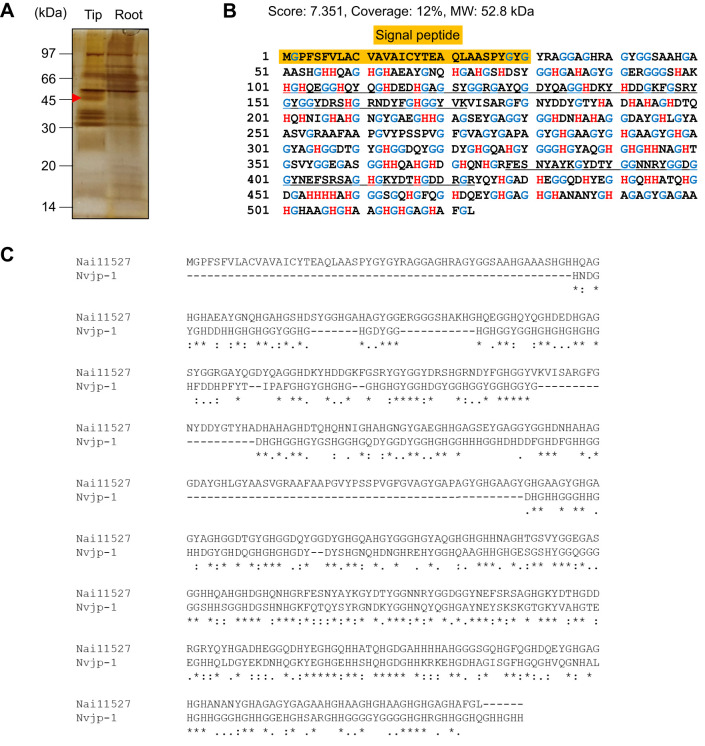
**Determination of Zn-binding protein in the jaw.** (A) SDS-PAGE of lower jaw extract (tip and root). The red arrow indicates the characteristic band in the tip, which was subjected to LC-MS/MS analysis. (B) Sequence of Nai11527 (sequences highlighted in orange are signal peptides; red indicates histidines; blue indicates glycines). (C) Sequence alignment of Nai11527 and Nvjp-1. The multiple alignment was conducted using MAFFT. Asterisks indicate positions that have a single, fully conserved residue. Fully conserved strong (‘:’) and weak (‘.’) groups are also indicated.

To identify the Zn-binding protein, the protein extracted from the jaw was subjected to SDS-PAGE. The jaw was divided into tip and root sides. The results of SDS-PAGE showed a tip-specific candidate protein band at approximately 45 kDa (Fig. 7A). As Zn was concentrated at the tip, this specific protein band seemed to be a Zn-binding protein. The protein band was separated and subjected to LC-MS/MS analysis. RNA sequencing of green worms was performed to generate the amino acid database for LC-MS/MS. LC-MS/MS identified the protein of Unigene294 (gene 11527) (Table S3; Fig. 7B). We named the protein Nai11527. Nai11527 has a signal peptide and is rich in histidine and glycine. By BLAST search, Nai11527 showed high homology with the histidine-rich jaw structural protein of *Alitta virens* (accession no. ACD31683, e-value 2e−11). The partial sequence of Nai11527 is about 53 kDa; 29% of the total amino acids residues were glycine and 16% were histidine in Nai11527.

## DISCUSSION

In this study, molecular phylogenetic analysis of *16S* rRNA demonstrated the green worm was *N*. *aibuhitensis*. *16S* rRNA is useful for identifying the species of green worm. It is difficult to determine the species name from the appearance (Tosuji and Sato, 2008). *Nereis aibuhitensis* is found primarily in estuarine and intertidal areas along the western Pacific coast. *Nereis aibuhitensis* ingests sediment and is a beneficial organism for environmental restoration (Gong and Zhang, 2022; He et al., 2022; Jørgensen et al., 2008). Because of their sediment-feeding nature, these worms are also good bioindicators for investigating organic and heavy metal pollution in sediments and estuaries (Zhao et al., 2020).

SEM analysis showed that the jaw of green worms had different structures at the tip and the root. The tip had a single-layer structure with organic matter, Zn and halogens. The root had a two-layer structure, with organic matter, Zn and halogens on the outside and only organic matter on the inside. The two-layer structure was similar to the jaw of *Nereis virens* (Birkedal et al., 2006).

The EDS analysis demonstrated that the structural elements were similar to those of *Nereis limbata,* consisting of Zn, Cl, Br and I. The inner part was composed of organic molecules and identified as jaw pulp. The hard outer tissue surrounds the pulp, protecting it from external stimuli and damage. Micro-CT observation revealed that elements with large atomic numbers are concentrated in the jagged, sharp-edged and curved regions. The hardening of these areas is thought to contribute to predation and migration. *Nereis virens* uses its jaws to grasp and tear prey, and Zn^2+^ coordinated by proteins is concentrated at the tips of the jaws and serrated edges, accounting for approximately 2% of the total dry mass (Broomell et al., 2006; Lichtenegger et al., 2003). Moreover, the edges of leaf-cutter ants' jaws exhibit higher levels of Zn. A correlation was shown between the amount of Zn and hardness (Schofield et al., 2002). EDS analysis showed that Br and I were abundant in the curved area of the *N*. *virens* jaw (Birkedal et al., 2006). Therefore, the presence of Br, I and Zn contributed to the brightness of these areas. PIXE analysis revealed that the localization of Zn and Cl overlapped at the center of the jaw. Cross-sectional observations also revealed that the localization of Zn and Cl was consistent with that observed in *N. limbata* (Lichtenegger et al., 2003). Based on the results of the elemental analysis described above, the following concept was suggested about the distribution of elements in the jaw. Br and I were concentrated in the jagged, sharp-edge and curved areas, which appeared as bright contrasts on micro-CT images. Zn was concentrated at the tip of the jaw and more abundant in the center than on the surface, consistent with the distribution of Cl. Although some variability was observed in the nanoindentation results, the data support the possibility that hardness may be enhanced in regions concentrated in Zn and Cl. Similar regional variations in mechanical properties have been reported for biological jaw materials, including those of the coffee borer beetle and *N. virens* (Broomell et al., 2008; Kundanati et al., 2020).

**Fig. 8.**
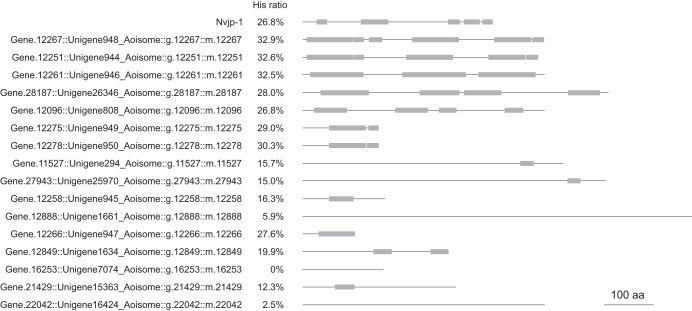
**Schematic comparison of histidine-rich domains among homologous proteins.** Horizontal bars indicate full-length amino acid sequences; gray boxes denote histidine-rich regions defined as either ≥10 consecutive His residues or ≥8 His residues within a 20-residue sliding window.

In the XAFS measurement for Zn, the spectrum showed a peak position intermediate between zinc halides and imidazole-Zn. These results suggest that Zn in the jaw exists as a mixture of halides and histidine-bound substances. The mineral phase of the jaw of *N. limbata* was not identified by XRD and XAFS analysis. Zn and Cl are known to bind to proteins and contribute to cross-linking within the protein matrix in the jaw of the *N. limbata* (Lichtenegger et al., 2003). Furthermore, Zn directly binds to Cl in *Nereis* jaws (Khan et al., 2006). In addition, XRD patterns in this study indicated that Zn in the jaw exists in a chemical form intermediate between imidazole ligands and halides. Several studies have reported that Zn binding to halogens and proteins contributes to increased jaw strength. The jaws of *N. virens* are probably encased in halogenated, cross-linked protein, Nvjp-1, expressed at the tip of the jaw of with Nai11527. Nvjp-1 is also rich in histidine and glycine (Broomell et al., 2008). Nai11527 and Nvjp-1 had high homology in the histidine-rich region in the second half of the sequence (Fig. 7C). BLAST analysis revealed multiple transcripts of *N*. *aibuhitensis* showing varying degrees of similarity to Nvjp-1 (Table 2). The domain structure of these proteins was schematically illustrated based on amino acid length and the position of histidine-rich regions, defined as either ≥10 consecutive histidine residues or ≥8 histidines within a 20-residue sliding window (Fig. 8), and their sequences are listed in Dataset 1. The highest alignment score was observed for Unigene948 (score=757, e-value=0), though its expression level was moderate (fragments per kilobase of transcript per million mapped reads, FPKM=503.68). In contrast, Nai11527 (Unigene294), with a lower similarity score (score=308), showed the highest expression level (FPKM=9672.48), suggesting a dominant functional role despite lower sequence identity. This pattern indicates that Nvjp-1-like functions may be distributed across a family of paralogous genes with diverse expression profiles and evolutionary divergence in this species. This combination of cross-linking and halogen bonding stabilizes the jaws, making them insoluble and less susceptible to chemical and enzymatic attack (Birkedal et al., 2006). Cross-linking of melanin with metal ions increased strength in the jaw of the Glycera family, which belongs to the same order as the Nereis family (Moses et al., 2006). The jaw of *N. aibuhitensis* may also use proteins to increase hardness of the tissue. Given the common characteristic of being histidine rich in these amino acid sequences, Zn at the tip of the jaw was bound to the nitrogen within the imidazole ring of histidine in Nai11527. In addition, the jaws were hardened by Zn connected with Nai11527.

**Table 2.** Sequence similarity and expression levels of Nvjp-1-like transcripts

	Score	e-value	FPKM
Gene.12267::Unigene948_Aoisome::g.12267::m.12267	757	0	503.68
Gene.12251::Unigene944_Aoisome::g.12251::m.12251	741	0	1158.8
Gene.12261::Unigene946_Aoisome::g.12261::m.12261	401	e−112	924.83
Gene.28187::Unigene26346_Aoisome::g.28187::m.28187	364	e−101	188.97
Gene.12096::Unigene808_Aoisome::g.12096::m.12096	330	3.0e−91	52.13
Gene.12275::Unigene949_Aoisome::g.12275::m.12275	312	1.00e−85	149.4
Gene.12278::Unigene950_Aoisome::g.12278::m.12278	308	2.00e−84	217.24
Gene.11527::Unigene294_Aoisome::g.11527::m.11527	308	2.00e−84	9672.48
Gene.27943::Unigene25970_Aoisome::g.27943::m.27943	284	3.00e−77	7728.83
Gene.12258::Unigene945_Aoisome::g.12258::m.12258	198	2.00e−51	13.54
Gene.12888::Unigene1661_Aoisome::g.12888::m.12888	88	5.00e−18	3.5
Gene.12266::Unigene947_Aoisome::g.12266::m.12266	87	1.00e−17	321.81
Gene.12849::Unigene1634_Aoisome::g.12849::m.12849	86	2.00e−17	3.61
Gene.16253::Unigene7074_Aoisome::g.16253::m.16253	68	5.00e−12	0.89
Gene.21429::Unigene15363_Aoisome::g.21429::m.21429	67	9.00e−12	1.83
Gene.22042::Unigene16424_Aoisome::g.22042::m.22042	66	1.00e−11	2.47

For sequence details, see Dataset 1. FPKM, fragments per kilobase of transcript per million mapped reads.

These results suggest two potential applications. The first is the development of hard materials with reduced metal content. Nanoindentation results indicated that regions concentrated in Zn and Cl exhibited significantly higher hardness than other regions. In this study, the jaw utilizes organic material to concentrate Zn, creating a strong structure. The jaw of *Glycera* species belonging to the Polychaete class has higher wear resistance than vertebrate dentin, despite having 1/17 the mineral volume fraction compared with dentin (Lichtenegger et al., 2002). Practically, adding Zn to vinylimidazole-based polymers to mimic the jaws of the *Nereis* species can increase tensile strength by a factor of 10 or more (Srivastava et al., 2008). This may lead to the development of special high-hardness materials using Nai11527.

The second potential application is the recovery of Zn from the environment. Gamma-glutamyl peptides, which bind various heavy metals in plants, algae and fungi, could be used for heavy metal recovery (Narender Reddy and Prasad, 1990). Similarly, proteins present in animals, such as Nai11527, are also expected to be used for metal recovery.

### Conclusion

Approximately 6% of zinc in the jaws of *N. aibuhitensis* was localized at the tip, predominantly on the inner side. The zinc appeared to be in a non-crystalline form and is probably bound to a histidine-rich protein, Nai11527. Our study has the potential to contribute to the development of hard materials with reduced metal content and to the recovery of zinc from the environment using the Zn-binding protein Nai11527.

## Supplementary Material

10.1242/jexbio.251316_sup1 Supplementary information
