# Supplementary material for: Zinc accumulation in the jaw of Nereis aibuhitensis
Source: J Exp Biol. 2026 Apr 16;229(8):jeb251316. doi: 10.1242/jeb.251316 (PMC13120676; doi:10.1242/jeb.251316)
Supplement: Supplementary information [file jexbio-229-251316-s1.pdf]

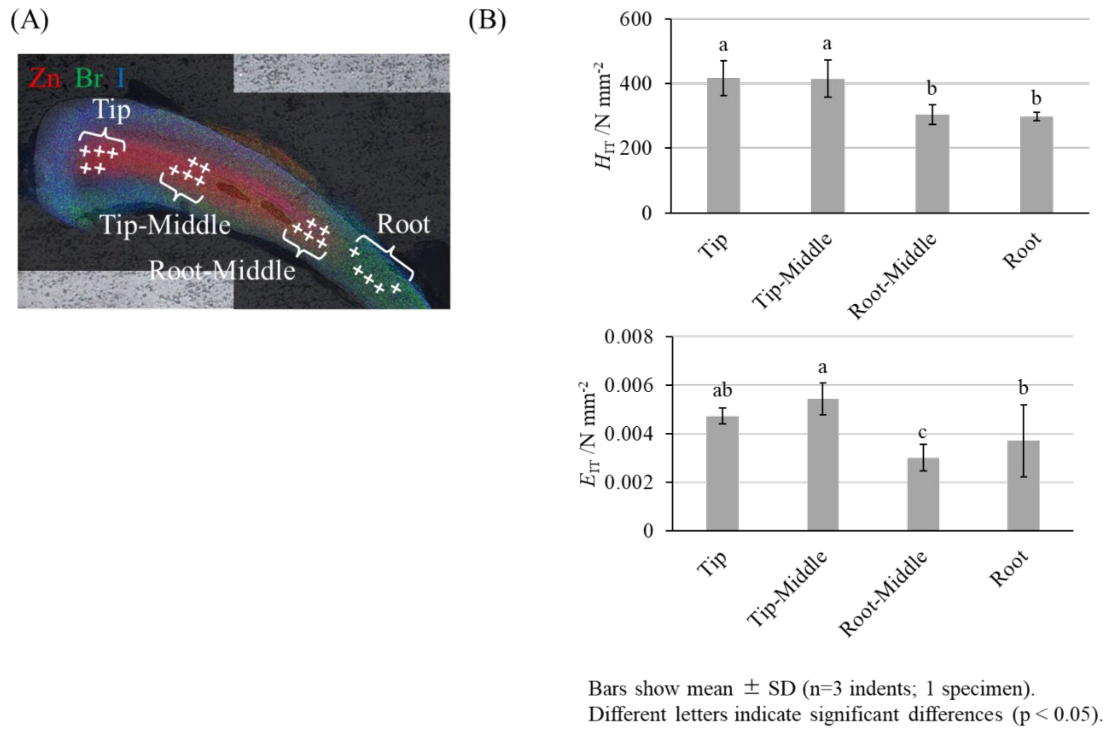

Fig. S1. Physical properties of center regions of the jaw (A) The area measured by nanoindentation. The colors correspond to the elements (red: Zn, green: Br, and blue: I), with brighter colors indicating higher elemental concentrations. (B) The nanoindentation hardness values (H_{IT}) and Young's modulus values (E_{IT}) of each region are illustrated in Fig. S1A (N = 5 indents; 1 specimen, mean s.d.). Statistical significance was determined using the Tukey–Kramer test. Different letters indicate significant differences ($p < 0.05$). The Tip region and Tip–Middle region correspond to Region 1 and Region 2 in Fig. 5, respectively.

Journal of Experimental Biology • Supplementary information

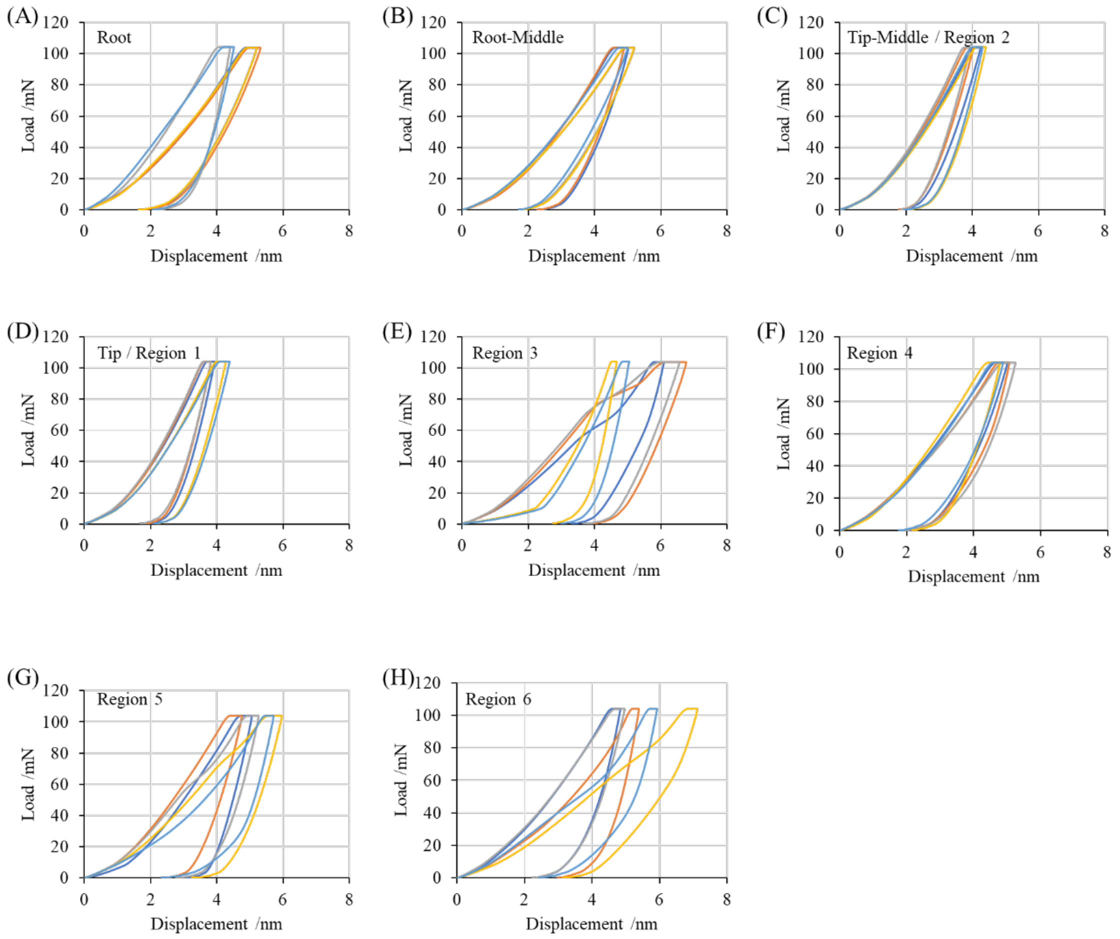

Fig. S2. Load–Displacement curves for each region shown in Fig. S1 (A–D) and for Region1–6 in Fig. 5 (C–H). For each region, three independent indentations (N=5 indents) are shown, with different colors representing individual measurements. The Tip and Tip–Middle region in Fig. S1 correspond to Region 1 and 2 in Fig. 5, respectively.

Journal of Experimental Biology • Supplementary information

Table S1. Neanthes family and Glycera family used for the phylogeenetic tree.

Accession number	Scientific name
KF483112.1	*Nereis quatrefagesi*
LC482191.1	*Nereis shikueii*
LC482189.1	*Nereis wilsoni*
LC482168.1	*Nereis mictodonta*
LC482160.1	*Nereis nuntia*
KC833498.1	*Nereis brevicirris*
KC833495.1	*Nereis cultrifera*
KC833486.1	*Nereis aibuhitensis*
KC833492.1	*Nereis heterocirrata*
AY340470.1	*Nereis pelagica*
AB703100.1	*Hediste diadroma*
LC323067.1	*Hediste japonica*
MF850380.1	*Neanthes wilsonchani*
MZ408682.1	*Neanthes goodayi*
HM746711.1	*Glycera tridactyla*
DQ779615.1	*Glycera alba*

Table S2. The weight of samples and BN for XAFS measurement

Sample name	Sample weight /mg	BN weight /mg
Nereidae	50.0	50.0
ZnO	11.1	88.9
$Zn(OH)_2$	11.8	88.2
$ZnCl_2$	15.4	84.6
$ZnBr_2$	23.1	76.9

Journal of Experimental Biology • Supplementary information

Table S3. Results of protein identification by LC-MS/MS

Description	Score	Coverage /%	MW /kDa
Gene.11527::Unigene294_Aoisome::g.11527::m.11527	7.351	12	52.8
Gene.10929::CL2611.Contig2_Aoisome::g.10929::m.10929	5.812	24	41.8
Gene.3827::CL706.Contig1_Aoisome::g.3827::m.3827	5.757	18	33
Gene.12261::Unigene946_Aoisome::g.12261::m.12261	3.537	7	50.6
Gene.12267::Unigene948_Aoisome::g.12267::m.12267	2.156	4	49.9
Gene.19271::Unigene11743_Aoisome::g.19271::m.19271	1.575	12	12.8
Gene.2207::CL391.Contig2_Aoisome::g.2207::m.2207	1.058	1	140.2
Gene.12404::Unigene1083_Aoisome::g.12404::m.12404	0.824	2	75.2
Gene.5808::CL1102.Contig1_Aoisome::g.5808::m.5808	0.749	3	35.9
Gene.1078::CL180.Contig2_Aoisome::g.1078::m.1078	0.731	1	76.9
Gene.14544::Unigene4218_Aoisome::g.14544::m.14544	0.535	7	17.2
Gene.25295::Unigene21667_Aoisome::g.25295::m.25295	0.535	3	49.3

Journal of Experimental Biology • Supplementary information

Dataset 1. Amino acid sequences of Nvjp-1–like proteins identified in green worm.

>Gene.12267::Unigene948_Aoisome::g.12267::m.12267
Gene.12267::Unigene948_Aoisome::g.12267 ORF type:5prime_partial len:485 (+)
Unigene948_Aoisome:2-1456(+)
HNDYGFKGGDKHYHHDDGHHKDHHYGHGGGHGGGEGYGHGGGHHHHHDDHGDDHHH
GGGH
EGHYGGGHDSHHGGHGHGHGHHDGHHSGHGEHDSGHHGGYGGHGGGHHDGYGHGGHH
GHG
HGGYGGHGHGGYVGHGHGGHGHGGHGHGGHGHGYHDHPFYAIPAYGHGHGHGGFGHGF
GG
HGGHGGFGHGGHGGYGHDDHGGHGYGGHGGHGYDQGGDYGGHGHGGHHHSGHDHDD
FGHD
FGHHGGDHGHHGGHHHGHHGGHGHDHGHGHGHGGYGYGHGDHHDHGHRDHHDGHHE
GGHH
GHGHHGSHYGGQGGGGGGHHSGGHDGSHNHGKFESHYSYKGHDKYGGHNKYHGHGAY
NEY
SKSKGTGKYVAHGAEEGHHDLTGYEKDHHKGDYEGHGEHHSHHHGDGHHKRKEHGDHA
GV
SGHHAHGHVQGHHGLQGHHGGGHGHHGGAHGHYARGHHGGGGHGGGGHGHSGHHGG
HHGHHGHH*

>Gene.12251::Unigene944_Aoisome::g.12251::m.12251
Gene.12251::Unigene944_Aoisome::g.12251 ORF type:5prime_partial len:473 (+)
Unigene944_Aoisome:2-1420(+)
HNDYGFKGGDKHYHHDDGHHKDHHYGHGGGHGGGEGYGHGGGHHHHHDDHGDDHHH
GGGH
EGHYGGGHDSHHGGHGHGHGHHDGHHSGHGEHDSGHHGGYGGHGGGHHDGYGHGGHH
GHG
HGGYGGHGHGDYGGHGHGGHGHGYHDHPFYAIPAYGHGHGGFGHGFGGHGGHGGFGHG
GH
GGYGHDDHGGHGYGGHGGHGYDQGGDYGGHGHGGHHHSGHDHDDFGHDFGHHGGDH
GHHG
GHHHGHHGGHGHDHGHGHGHGGYGYGHGDHHDHGHRDHHDGHHEGGHHGHGHHGSH
YGGQ

Journal of Experimental Biology • Supplementary information

GGGGGGHHSGGHDGSHNHGKFESHYSYKGHDKYGGHNKYHGHGAYNEYSKSKGTGKYV
AH
GAEEGHHDLTGYEKDHHKGDYEGHGEHHSHHHGDGHHKRKEHGDHAGVSGHHAHGHV
QGH
HGLQGHHGGGHGHHGGAHGHYARGHHGGGGHGGGGHGHSGHHGGHHGHHGHH*

>Gene.12261::Unigene946_Aoisome::g.12261::m.12261
Gene.12261::Unigene946_Aoisome::g.12261   ORF type:3prime_partial len:487 (+)
Unigene946_Aoisome:73-1530(+)
MKLVAALATAAILSLAVQAQHHGHHGHGGHGGHHGGHGHHGHHHGGGHGHGGHAHGGY
HG
HGGGGGHHGGHHGHHGGVSGHHHLTGHEAHGGHKHDQGHKGHHQHGHVQSHHNYAGH
EKG
HGDGDYGQHGGYKGHHHDHGHDNYNDDGKFNSHYGYDGYDHNEGSNEYFGHGGYVKV
VES
KGHGQYDDFGNYHNDYGFKGGDKHYHHDDGHHKDHHYGHGGGHGGGEGYGHGGGHH
HHHD
DHGDDHHHGGGHEGHYGGGHDSHHGGHGGAHGHHDGHHHGHGGHDSGHHGGYGGHG
HGHH
DGHGHHDHHGHGHGGYGGHGHGHYDDHPFHAIPAYGHGHGGHGHGYGGHGGYGHDDH
GGH
GYGDHGGHGHHHGGEYGGHGHGGHHHGGHDHDEFGHDFGHHGGDHGHHGGHHHGHH
GGHG
YDHGHGHGHGGYGYGHGDHHDHGHKDHHDGHQEGGHHGHGHHGSHYGGQGGGGGH
HSGG
HDGSHN

>Gene.28187::Unigene26346_Aoisome::g.28187::m.28187
Gene.28187::Unigene26346_Aoisome::g.28187   ORF type:5prime_partial len:615 (+)
Unigene26346_Aoisome:3-1847(+)
HFQRSCFQPNLRPGMKVALKLAGLLAISLVIFEAAAVHSGYPHGYGHHHGDHGHHGEHGH
HGGHGHEGYGHHGHAHGGAHGHDHGHGHHGGHAHYGHHGGHEGHHHGGGHGGHHEH
GHDK
EHYDYKGHHDHKGDGHYGGHGKHESDYHDHGHDKYDDDGKFDSHYGYKGYDKNKGHN
AYY

Journal of Experimental Biology • Supplementary information

GHGGYVKVVSAKGYGNYDDYGNYHMKHGHHGYEKDYDHNKHYGHDKSYGHSKGYGH
DDHY
GHHEGHGKHYGHDHGHDDYHHDHHDGDYGKHHDYDHGYGHDDHHGHDKHYGHDDHH
YGHD
DGYHHDHDHDDYGHGHDGHDGYDDHKHYAVAPYGHHHGHGHGHGDYGHGHGYGHDH
GYGH
GHGHGHGYGHDDHHHGHGHDHYGHKGYGHHGHGHGYHDKNKYGHGHKYGHEQGYGH
HDHD
KHGGEYGGHGEHHTYHKGHGEEGLKNYGKYQTHYAYKGYDTYGGHNHYDGVGAYNEYA
KS
KGYGKYQKHGEEEGYHKHHGYDKHDGHSDYKGHGEHHGYHKGGGHHDKHDYGKHEGH
HGA
HGYAHSHGGAHHDGHYGHKHGHDAHGHGHHEGYGHGHHDKHGHHGGHDHHKSYDIPP
EVT
TDTPTTTTTKQSEE*

>Gene.12096::Unigene808_Aoisome::g.12096::m.12096
Gene.12096::Unigene808_Aoisome::g.12096 ORF type:complete len:487 (-)
Unigene808_Aoisome:380-1840(-)
MAWTTRQRCALVLLLAITASAQYHEGYGHGHHSHGQHGHGDHQHYGGHKDYGAAGGHH
GH
HHGGGHEGHHEKGHAHDQYHAKGHTDHHVDGHYGGHGSHASDYHNHGHDKYDDDGHF
DSH
YGYKGTDQFKGRNQYYGQGGYVKVVSAKGYGNYDDYGQYHMEHGHKGYDKHYDHDK
HYKH
DKHYGHDKHYGHDDHYGHDYGHNHHDHHGHGHDDYGHDHDHYGHHGHDDYGHGHHD
GYGH
HDGYGHHHDDDKYYAPAPYGHYHNGYDHGYGKKNDYGHHGYGHDHGHDHYGHHGHG
HEGY
GHGYGHGHDYGHGHGYGQEHGYGHHDHDKHGGEYGGHGHKSGYHKGHGHDGHRNYG
KYET
HYAYKGYDDYGGKNHYDGYGGYNEYAMSKGYGKYDKHGGDKGYHQHHGYDKAKAHS
DHHG
EGHHHTYKHGDGHHDHHDYGKQQEHHDKHAYQKGYGSEGHHAHHGHHDHQDHHGHGH
HGN
HGHHGY*

>Gene.12275::Unigene949_Aoisome::g.12275::m.12275

Gene.12275::Unigene949_Aoisome::g.12275 ORF type:5prime_partial len:153 (+)

Unigene949_Aoisome:1-459(+)

FESHYSYKGYDKYGGHNKYHGHGAYNEYAKSKGTGKYIAHGAEEGHHELNGYEKDHHKG

D

YEGHGEHHSHHHGDGHHKHKEHGDHAGASGHHAHGHVQGHHGLQGHHGGGHGHHGGA

HGH

YARGHHGGGGYGGGGHGHSGHHGGHQGHHGHH*

>Gene.12278::Unigene950_Aoisome::g.12278::m.12278

Gene.12278::Unigene950_Aoisome::g.12278 ORF type:5prime_partial len:153 (+)

Unigene950_Aoisome:1-459(+)

FESHYSYKGYDKYGGHNKYHGHGAYNEYAKSKGTGKYIAHGAEEGHHELNGYEKDHHKG

D

YEGHGEHHSHHHGDGHHKHKEHGDHAGASGHHAHGHVQGHHGLQGHHGGGHGHHGGA

HGH

YARGHHGGGGHGGGGHGHSGHHGGHHGHHGHH*

>Gene.11527::Unigene294_Aoisome::g.11527::m.11527

Gene.11527::Unigene294_Aoisome::g.11527 ORF type:complete len:524 (+)

Unigene294_Aoisome:23-1594(+)

MGPFSFVLACVAVAICYTEAQLAASPYGYGYRAGGAGHRAGYGGSAAHGAAASHGHHQA

G

HGHAEAYGNQHGAHGSHDSYGGHGAHAGYGGERGGGSHAKHGHQEGGHQYQGHDEDH

GAG

SYGGRGAYQGDYQAGGHDKYHDDGKFGSRYGYGGYDRSHGRNDYFGHGGYVKVISARG

FG

NYDDYGTYHADHAHAGHDTQHQHNIGHAHGNGYGAEGHHGAGSEYGAGGYGGHDNHA

HAG

GDAYGHLGYAASVGRAAFAAPGVYPSSPVGFGVAGYGAPAGYGHGAAGYGHGAAGYGHG

A

GYAGHGGDTGYGHGGDQYGGDYGHGQAHGYGGGHGYAQGHGHGHHNAGHTGSVYGGE

GAS

GGHHQAHGHDGHQNHGRFESNYAYKGYDTYGGNNRYGGDGGYNEFSRSAGHGKYDTHG

DD

Journal of Experimental Biology • Supplementary information

RGRYQYHGADHEGGQDHYEGHGQHHATQHGDGAHHHHAHGGGSGQHGFQGHDQEYGH
GAG
HGHANANYGHAGAGYGAGAAHGHAAGHGHAAGHGHGAGHAFGL*

>Gene.27943::Unigene25970_Aoisome::g.27943::m.27943
Gene.27943::Unigene25970_Aoisome::g.27943 ORF type:5prime_partial len:610 (-)
Unigene25970_Aoisome:512-2341(-)
TQVSAHLSGIGKTVTMGPTLLLFFTAAVAVSNAGPGAPSLSPYGYGYRGGAGHAAGYGGA
AGFGANAAHGHHQAGHGHAEAHGNQHGAYGGHDAYGGHGQHAGYGGSQGGGGHAQH
GHTE
GGHQYQGHDEDHGKGHYGGSGAYQGDYQAGGHDKYHDDGQFGSRYGYGGYDRSHGRN
DYF
GHGGYVKTISARGYGNYDDFGQYHTDHAHAGHDTQHQHNIGHAHGQGYGTEAHHGAGA
DY
GQGAYGGHDDYAHGHGDAHGYAGNDYGHGIAHGHAGNAFADNFVARAIPAYGLGRSHHG
D
YNGGLYGYGRPKDRGIANFGRGYHTGSGFGVFGRSYAIPAYPGPAPLPAPAADPYGVGLA
PHGAGYGHGAGYAGHGAGYGHGASYAGHGADANYGHGGDHYGGDYAHGAGHGYGGG
HGYA
QGHGHGHHNAGHTGSVYGGEGASGGHHQAHGHDGHQNHGRFESNYAYKGYDTYGGNNR
YG
GDGGYNEFSRSRGHGKYDTHGDDRGRYQYHGADHEGGQDHYEGHGQYQATQHGDGGHH
HH
AHGGGSGQHAHHGHDQTYGHGAGHGHANANYGHAGAGYGAAATHGHAAGHGHAAGH
GHGA
GYGHAAGLP*

>Gene.12258::Unigene945_Aoisome::g.12258::m.12258
Gene.12258::Unigene945_Aoisome::g.12258 ORF type:5prime_partial len:167 (+)
Unigene945_Aoisome:1-501(+)
FESHYSYKGYDKYGGHNKYHGHGAYNEYAKSKGTGKYIAHGAEEGHHELNGYEKDHHKG
D
YEGHGEHHSHHHGDGHHKHKEHGDHAGVSGHHAHGHVQGHHGLQRTPRRRSRTSWRSS
WS
LRQRSSWWWRTWWRWPRSQWPPWRSSWSPWPSLVLKMQLDYTRLFH*

Journal of Experimental Biology • Supplementary information

>Gene.12888::Unigene1661_Aoisome::g.12888::m.12888

Gene.12888::Unigene1661_Aoisome::g.12888 ORF type:internal len:783 (+)

Unigene1661_Aoisome:1-2346(+)

IRSAPNRGYRMPPFSMVNLTHGIRKELVRMGHVSGHKPTAVVKPQESSYSYATNKPHRSQ

DYQYDRRQDNYNDGYRQNNYQRYSDYRGGHSDRRYEQNDRRYNQNDRLFDQNDRRYED

RY

DDRQRGRYNQHGGANNNANYKGGAYDRDQRYNQNRAKQLKGEDDFAKFWQELEEGVKF

DS

SKKEPAKFVQEKSNQPSLSQAAKALPNTFEPNWMKDTKKAANQSEQASQAASQSNDLIQQ

LSQQLLSDLSVNSQSGQEVLLSKMKSLEMSNKVAAAASDPQVFCDPAILVTGSSESKAKV

ANDKDGTAALQDLLKIGQHKNEPAKSEATQQPQYSKQVSLQELLNGYQPTAEPSQQQPYQ

QHQQPQQHQQPQHQQQQQHQQQQQRKQQQPQQNKQQGKQGGKRNRVNELKTFVQALG

VPA

PQFNLSDTDNGGYVATVILSNGQRFQGSNAWKQEQALESAASVALLNLQGGGQTMPPFMM

GVQPQQPHRHGNHPHQQQQQQHQQQQRFQGPRGNFSSSPNSAFTPVKPSGKGPQPFFDP

RNNVQNVNFSSPPQHRPSGPPHQQHAPPQGWSPHGMPGNHGPRGTFNPQGRHGNYGNHPG

NHPGNHPGNHPANHPGNHPGNQGHQFGPHHGNQGGSHHGGHYHGNQGYHGNQGHYGNQ

GG

HYGNQGHHHGNQGHQGYHDHRNRQLSESGRGQSQEKQNYDGQDGEGTPGKNPFIPLQVQ

R

KQKTPQRDVKKRLEDSYNNGDASHSKRDSSKSGGKSKDSETLEGYAKSEKKSGSDQQRRE

HK

>Gene.12266::Unigene947_Aoisome::g.12266::m.12266

Gene.12266::Unigene947_Aoisome::g.12266 ORF type:internal len:106 (+)

Unigene947_Aoisome:1-315(+)

VNPGKHPPLDSSVQTREMKLVAALVTAAILSIAVQGQHHHGHHGGGHGGHHGGHGHHGHH

HGGGHGHGGHAQGGYHGHGGGGGHHGGHHGHHGGVSGHHHLTGQE

>Gene.12849::Unigene1634_Aoisome::g.12849::m.12849

Gene.12849::Unigene1634_Aoisome::g.12849 ORF type:internal len:293 (-)

Unigene1634_Aoisome:1-876(-)

VMAVCYVRKKAAMIKVIHCHLRCCLLSLLLMQMSHCAPDDSNFHRHDHTGAVQDAVNPN

N

LPEDSKMGISLAQSATRKEAELFLDHLFVKYGRKGVMTFEGFEHLLQSLGIGNVTIVDHD

Journal of Experimental Biology • Supplementary information

LKDHYTESGFVEFHEDHRHVLKIEDDHHDHSHDDHDHDHSHHDDNHHGSHHHGSNADDD
N
GNYDVGRNRSGYNQSGSLRHGHDSNGHRNHNHNHGNSDSNHNGHNHDSHDHDGDHGNH
RV
KQGHDHSSHGNRDRDDNHDHHDHNEHHKKDKFGHSHEVHHQDHYDHDHSLHH

>Gene.16253::Unigene7074_Aoisome::g.16253::m.16253
Gene.16253::Unigene7074_Aoisome::g.16253 ORF type:internal len:163 (+)
Unigene7074_Aoisome:1-486(+)
GPGTGGYGAGGPGSGLGGAGTPGSGVDDMGAGGPGGYGAGGPGRPGSGVGAGGPEGYG
GP
GAGGPGAGGPGAGGPGTGGPGAGGPGAGGYGAGGPGSGIGGVGAGGPGSGAGGPSSGAG
G
PGSGAGGPGAGGPGSGIGGLGVGGPGSGAGGPGAGGPGGFGP

>Gene.21429::Unigene15363_Aoisome::g.21429::m.21429
Gene.21429::Unigene15363_Aoisome::g.21429 ORF type:5prime_partial len:309 (+)
Unigene15363_Aoisome:1-927(+)
TLSQENNTTNVTKADNDDSNFLYFWDDEDYDDYDQDPEGQIEGHGDHQGHDGHHQEHEV
T
GDDKARHQHHGNHHKHHGQQQNDHKDHHNHQNDHTDHQNHQNHMDKQNDHTGHQND
NTGQ
MNHHGKGGPKSDHQNFQNEQSHHVSDGHQNNDASYVMDNGHGMHGVGHDMGHTMKM
YFHA
GVDAVVLLKEWTITSGTGMVGSCVILIIISTLYEGLKVFKDRINLSCQDKKQCCSCCSCL
PARMCRGVHFLQTLVHVLQVFISYLLMLVFMTYNIYLCLSVLLGTGLGYFFFRYNSHDRV
ESDEDHCN*

>Gene.22042::Unigene16424_Aoisome::g.22042::m.22042
Gene.22042::Unigene16424_Aoisome::g.22042 ORF type:internal len:487 (+)
Unigene16424_Aoisome:3-1460(+)
KSDYNRSQDYGRHDYGRHNEYGRGQDYGRQHDYGGRDYNNYRGQDYGGRNNDYGNSN
HDY
NSKGYDYGNRNQEYNRNMDYSNRNQDYSNRNYEYSNKRSQGPRGGRGQENSEDNDRRG
GK
NYNEGRQRGSHNNQEEKTEKAPRYNASSQLADGDPSIVSVNQQPPQPKPAAGPAAIKLDH

Journal of Experimental Biology • Supplementary information

YGPPPSAAAILTSTTQPGYSASMPNSQVQMNPLPSQPAVPPPNHIQPVYSPPEGSMVSEG
QTVVYTSPQTLQYSSTPLVNQQLIQQVQDLSLVSSGYSVQPHGEPVMTSVPHGVQPQTMQ
FNSVARPDKVKHAAENRVMRHWKPGDFCMARYWHDGKYYRAMIQAVVQNGQGCMVTFP
DY
GNTERVPLSDVRAIPKQAWESNSIQGQVMDMIPTVPATGAPTGMTAQTVINPGMVPVSMP
APAMIVPTSPTMTYVTYPSQAPPTDQSYQGQITGLEFYQSGGGIAYQMNAAPRNNQRPAQ
QYYLQT

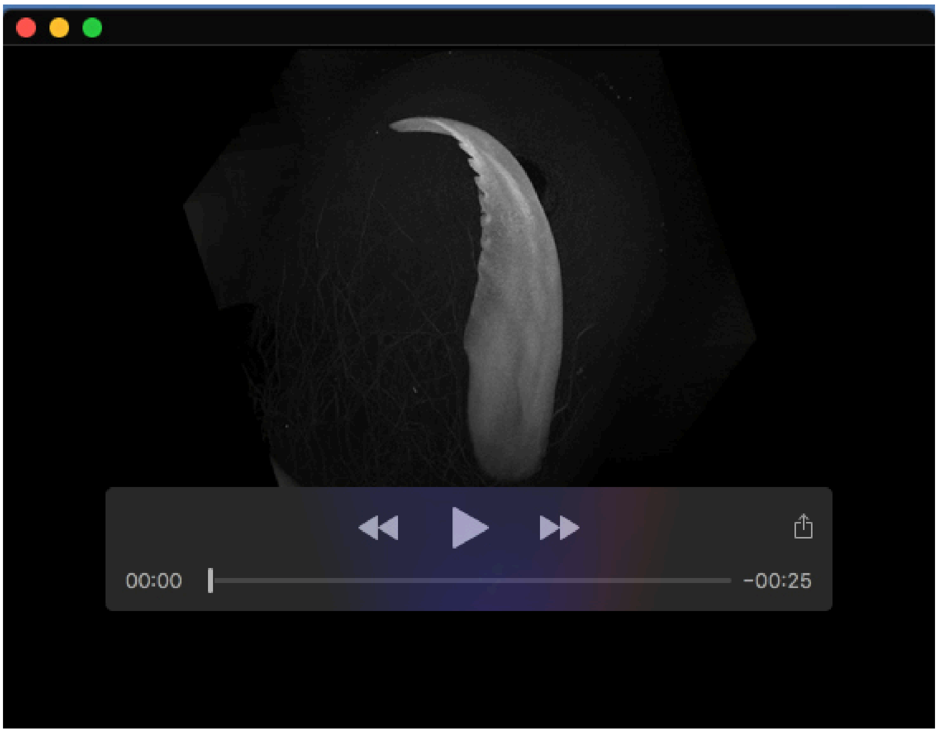

Movie 1. Three-dimensional reconstruction of the jaw obtained by micro-CT.
